# Supplementary material for: Pyruvate Dehydrogenase Kinase 1 inhibition mediated oxidative phosphorylation enhancement in cartilage promotes osteoarthritis progression
Source: BMC Musculoskelet Disord. 2023 Jul 20;24:597. doi: 10.1186/s12891-023-06585-6 (PMC10357736; doi:10.1186/s12891-023-06585-6)
Supplement: Supplementary file 1 — Additional file 1. [file 12891_2023_6585_MOESM1_ESM.zip › KEGG.Gsea.1653622432464/KEGG_ALPHA_LINOLENIC_ACID_METABOLISM.html]

Details for gene set KEGG\_ALPHA\_LINOLENIC\_ACID\_METABOLISM[GSEA]

|  || Dataset | 55457\_collapsed\_to\_symbols.cls.cls #OA\_versus\_normal.cls.cls #OA\_versus\_normal\_repos |
| Phenotype | cls.cls#OA\_versus\_normal\_repos |
| Upregulated in class | normal |
| GeneSet | KEGG\_ALPHA\_LINOLENIC\_ACID\_METABOLISM |
| Enrichment Score (ES) | -0.22249964 |
| Normalized Enrichment Score (NES) | -0.63414514 |
| Nominal p-value | 0.9338235 |
| FDR q-value | 1.0 |
| FWER p-Value | 1.0 |
Table: GSEA Results Summary

  

Fig 1: Enrichment plot: KEGG\_ALPHA\_LINOLENIC\_ACID\_METABOLISM      
 Profile of the Running ES Score & Positions of GeneSet Members on the Rank Ordered List

  

| SYMBOL | TITLE | RANK IN GENE LIST | RANK METRIC SCORE | RUNNING ES | CORE ENRICHMENT || 1 | PLA2G12A | phospholipase A2 group XIIA [Source:HGNC Symbol;Acc:HGNC:18554] | 600 | 0.623 | 0.1056 | Yes |
| 2 | ACOX1 | acyl-CoA oxidase 1 [Source:HGNC Symbol;Acc:HGNC:119] | 1401 | 0.443 | 0.1517 | Yes |
| 3 | PLA2G4A | phospholipase A2 group IVA [Source:HGNC Symbol;Acc:HGNC:9035] | 2656 | 0.284 | 0.1239 | No |
| 4 | PLA2G10 | phospholipase A2 group X [Source:HGNC Symbol;Acc:HGNC:9029] | 3315 | 0.220 | 0.1265 | No |
| 5 | PLA2G3 | phospholipase A2 group III [Source:HGNC Symbol;Acc:HGNC:17934] | 3648 | 0.190 | 0.1472 | No |
| 6 | FADS2 | fatty acid desaturase 2 [Source:HGNC Symbol;Acc:HGNC:3575] | 5025 | 0.071 | 0.0578 | No |
| 7 | PLA2G2D | phospholipase A2 group IID [Source:HGNC Symbol;Acc:HGNC:9033] | 5056 | 0.069 | 0.0723 | No |
| 8 | ACOX3 | "acyl-CoA oxidase 3, pristanoyl [Source:HGNC Symbol;Acc:HGNC:121]" | 6747 | -0.059 | -0.0444 | No |
| 9 | PLA2G5 | phospholipase A2 group V [Source:HGNC Symbol;Acc:HGNC:9038] | 6772 | -0.061 | -0.0314 | No |
| 10 | PLA2G2E | phospholipase A2 group IIE [Source:HGNC Symbol;Acc:HGNC:13414] | 7486 | -0.117 | -0.0581 | No |
| 11 | PLA2G1B | phospholipase A2 group IB [Source:HGNC Symbol;Acc:HGNC:9030] | 8232 | -0.173 | -0.0736 | No |
| 12 | PLA2G2A | phospholipase A2 group IIA [Source:HGNC Symbol;Acc:HGNC:9031] | 10152 | -0.336 | -0.1403 | No |
| 13 | PLA2G2F | phospholipase A2 group IIF [Source:HGNC Symbol;Acc:HGNC:30040] | 10628 | -0.386 | -0.0829 | No |
| 14 | PLA2G6 | phospholipase A2 group VI [Source:HGNC Symbol;Acc:HGNC:9039] | 11128 | -0.446 | -0.0125 | No |
| 15 | JMJD7-PLA2G4B | JMJD7-PLA2G4B readthrough [Source:HGNC Symbol;Acc:HGNC:34449] | 12076 | -0.614 | 0.0639 | No |
Table: GSEA details [plain text format]

  

Fig 2: KEGG\_ALPHA\_LINOLENIC\_ACID\_METABOLISM      
 Blue-Pink O' Gram in the Space of the Analyzed GeneSet

  

Fig 3: KEGG\_ALPHA\_LINOLENIC\_ACID\_METABOLISM: Random ES distribution      
 Gene set null distribution of ES for **KEGG\_ALPHA\_LINOLENIC\_ACID\_METABOLISM**
